# Genetic variability analysis of Russian cultivars of ryegrass (*Lolium*) based on SCoT markers

**DOI:** 10.1186/s43141-022-00446-w

**Published:** 2022-12-13

**Authors:** Yulian Mavlyutov, Sergey Kostenko, Anastasia Shamustakimova, Irina Klimenko

**Affiliations:** Federal Williams Research Center of Forage Production & Agroecology (FWRC FPA), Lobnya, Moscow region Russia

**Keywords:** Ryegrass, Genetic diversity, SCoT markers, DNA polymorphism, Genotyping

## Abstract

**Background:**

Ryegrass is a promising crop for the development of meadow farming in the world. More than 1000 cultivated varieties widely used in feed production have been developed, based on the main species — perennial ryegrass (*Lolium perenne* L.) and annual one (*Lolium multiflorum* Lam.). Development and implementation of the modern methods of plant varietal and species identification are of great importance. In recent years, molecular markers have been successfully used for these purposes, which increase the accuracy of the breeding material evaluation at a significant reduction of time and labor costs. The aim of this study was to assess the discriminatory potential of the new SCoT marking technique for the identification of Russian perennial (*Lolium perenne* L.) and annual (*Lolium multiflorum* Lam.) ryegrass varieties.

**Results:**

Out of the total number of the tested SCoT-primers, 8 polymorphic ones were selected, which demonstrates the high stability and reproducibly amplified DNA fragments. These primers generated 107 PCR products, where 37 were found to be polymorphic. The average number of amplicons per primer was 13. The size of the PCR products varied from 349 to 2718 bp (see Table 3). The polymorphic ratio of the tested markers was 30.8%. The marker SCoT-06 was characterized by the maximum number of PCR products and the highest level of polymorphism (50%). The effective number of alleles (ne) ranged from 1.35 to 1.58 with a mean of 1.48 per locus. The average value of the PIC and Shannon index (I) were 0.35 and 0.46, respectively. The unique PCR fragments were revealed for the identification of tested varieties. Analysis of molecular variance (AMOVA) showed that the level of genetic diversity between ryegrass species (59%) was more than between varieties within a species (41%). Based on binary matrix data, clustering and PCoA analysis (see Figs. 1 and 2) of the samples were carried out that divided them into two groups according to species.

**Conclusions:**

We found a set of markers that can be useful tools for ryegrass varieties identification. The level of intravarietal polymorphism turned out to be higher than the differences between varieties because of the possible significant heterogeneity of the varietal material. The information obtained can be used in breeding programs to create improved ryegrass varieties adapted to Russian climatic conditions.

## Background

Perennial grasses are the main plants in natural meadow phytocenoses of the middle, north, and steppes of Russia, and are also used to create seeded meadows and pastures in modern agriculture. The widespread distribution of cereal grasses is due to their valuable biological properties — ecological plasticity, high productivity, longevity, winter hardiness, and vegetative regeneration. When grown in single-species crops and in grass mixtures with a legume component, they provide low-cost and high-quality forage (green and conserved in the form of silage and hay) and are used for landscaping, recultivation, and soil protection from water and wind erosion [1].

Two of the most promising crops in the worldwide practice of grassland culture are perennial (*Lolium perenne* L*.*) and annual (*Lolium multiflorum* Lam.) ryegrass. On the basis of these species, more than 1000 cultivars have been created that are of great importance for fodder production and the development of animal husbandry in many countries [2].

In Russia, the share of perennial ryegrass within the total crop structure of perennial grasses is about 6% [3]. Most cultivars were obtained using methods of population genetics, in particular, methods of creating synthetic hybrid populations and polyploidy. Russian ryegrass cultivars are distinguished by longevity (up to 6 years with 4–5-fold mowing), winter hardiness, increased shoots, short vegetative shoots, high productivity of dry matter (up to 11 t/ha), seeds (up to 0.8 t/ha), Intensive breeding work is being carried out with the aim of increasing productive longevity, resistance to fungal diseases, and creating specialized cultivars that allow for growing in mixtures with legumes [4].

Different ryegrass species usually have a diploid set of chromosomes (2n = 2x = 14), however, improved tetraploid cultivars are common [2, 5]. The cross-pollination system results in high heterogeneity within species and in individual populations and cultivars [6]. In this regard, research on the study of the genetic diversity of a crop is of particular importance, as a result of which it is possible to identify promising material for breeding, significantly reduce the time for breeding new cultivars and ensure copyright protection of breeders.

With the introduction of modern DNA technologies, new opportunities have emerged to improve the accuracy of source evaluation and selection efficiency. Various types of PCR-based molecular markers are widely used to study representatives of the genus *Lolium* L. In particular, Bolaric et al. [6] analyzed 22 European cultivars of pasture ryegrass using RAPD markers and found a higher degree of genetic polymorphism within cultivars compared to the level of intervarietal differences. High intravarietal genetic variability (81.99%) was characteristic of 6 Chinese annual ryegrass cultivars when analyzed using microsatellites [7]. ISSR markers were found to be effective in assessing the genetic variability of 50 samples of different ryegrass species [8]. Using SRAP markers, the genetic polymorphism of annual ryegrass cultivars from the USA, China, and Denmark was studied with an average value of over 89% [9].

In recent years, the SCoT (start codon targeted polymorphism) technology has been successfully used to differentiate and identify species and cultivars of cereal grasses. The high variability of markers provides a special structure of 18-nucleotide primers designed to amplify sequences in the genome flanking the ATG start codon [10]. The main advantages of this technique are that the synthesis of primers does not require prior information about the sequence being investigated, markers are aimed at the functional regions of the genome, and the results are highly reproducible [11]. Using markers of this type, the genetic diversity of cat grass was studied (*Dactylis glomerata* L.) [12], as well as the phylogenetic relationships between 19 species of the genus *Bromus* L*.* [13]. Also, these markers were used for genotyping hybrids of reed fescue and Italian ryegrass [14].

In Russia, at the moment, forage grasses remain a poorly studied area of molecular genetic research. The breeding of this group of plants is based, in most cases, on the phenotypic labeling of natural genetic variations between and within ecotypes. This approach causes certain difficulties due to the great variability of traits and properties, the complexity of the genetic system, and a high degree of plasticity in the interaction of the genotype and environmental conditions. Until now, Russian ryegrass cultivars are distinguished exclusively by morphological characteristics, which are often similar, despite the high genetic heterogeneity of culture. As a result, the accuracy of assessing the purity of varietal material is reduced, and the breeding process is laborious and time-consuming. The widespread introduction of molecular labeling methods will make it possible to choose the most suitable breeding strategy for the development of cultivars for various purposes, adapted to certain environmental conditions.

The aim of this study was to assess the potential of the new SCoT marking technique for the identification of Russian cultivars of perennial *(Lolium perenne* L.) and annual (*Lolium multiflorum* Lam.) ryegrass.

## Methods

### Plant material

The object of the study was 8 Russian ryegrass cultivars created by breeders of the Federal Williams Research Center of Forage Production & Agroecology (FWRC FPA) (Russia, Moscow region). To isolate DNA, we used “bulk samples” formed from 30 seedlings of each cultivar grown in Petri dishes for 7 days (Table 1).

**Table 1:** The studied cultivars of perennial and annual ryegrass

Cultivar (abbreviation)	Species	Ploidy	Originator
Agat (AG)	Perennial ryegrass (*Lolium perenne* L.)	4n	FWRC FPA
Duet (DT)		4n	FWRC FPA, State Unitary Enterprise “Moscow Breeding Station”
VIK 66 (V6)		4n	FWRC FPA
Karat (KT)		4n	FWRC FPA
Fenix (FX)		4n	FWRC FPA
VIK 22 (B2)		4n	FWRC FPA
Rapid (RD)	Annual ryegrass (*Lolium multiflorum* Lam.)	4n	FWRC FPA, State Unitary Enterprise "Moscow Breeding Station"
Moskovsky 74 (M74)		2n	FWRC FPA

### DNA purification

Genomic DNA was isolated by the SDS method [15] with some modifications, taking into account the type of plant tissue and the formation peculiarities of the sample (“bulk method”) [16]. Thus, the volume of the SDS extraction buffer was calculated individually for each sample: 300 ml per 30 mg of plant tissue; additional processing of extracts with RNAse was introduced, followed by incubation for 1 hour at 60 °C; excluded the stages of washing the extracts with solutions of phenol and chloroform. The quality and quantity of the obtained DNA were determined using electrophoresis in 1% agarose gel and a Nabi Nano Spectrophotometer (MicroDigital Co., Ltd., Korea). The final concentration of DNA solutions after dilution was 30 ng/μl.

### SCoT analysis

The studied ryegrass cultivars were genotyped using 25 SCoT markers (Table 2).

**Table 2:** SCoT markers for PCR analysis of ryegrass cultivars

No.	Primers	References
1	SCoT 01	[14]
2	SCoT 02	
3	SCoT 20	
4	SCoT 23	
5	SCoT 31	
6	SCoT 06	
7	SCoT 13	
8	SCoT 21	
9	SCoT 32	
10	SCoT 15	
11	SCoT 28	[17]
12	SCoT 11	
13	SCoT 26	
14	SCoT 08	[17]
15	SCoT 07	
16	SCoT 63	
17	SCoT 60	
18	SCoT 59	
19	SCoT 22	[18]
20	SCoT 36	
21	SCoT 35	[13]
22	SCoT 40	
23	SCoT 44	[12]
24	SCoT 17	[10]
25	SCoT 29	

The total volume of the PCR mixture was 10 μL and contained 1x Taq Turbo buffer, 1x dNTP mix (250 μM of each deoxynucleotide in the final volume), 0.5U Taq DNA polymerase, 0.8 μM primer, and 30 ng DNA. The reagents that are used are produced by the Russian company Evrogen. Amplification was carried out on a T100 Thermal Cycler device (Bio-Rad, USA) at the following mode: 3 min – 94°C; then 35 cycles of 1 min – 94°C, 1 min – 50°C, 1 min – 72°C; and 5 min – 72°C. A 1.6% agarose gel was used to separate the amplification fragments.

### Data analysis

The size of the obtained PCR products was determined using the Image Lab version 6.0.1 software package on a GelDoc XR Plus device (Bio-Rad, United States) in comparison with a 1kb DNA Ladder molecular weight marker (Evrogen, Russia). Based on the data obtained, a binary matrix was formed where the presence of the product was designated as “1,” and the absence – “0”. In the analysis only distinct and reproducible bands were considered.

The POPGENE program of version 1.32 was used to calculate the effective number of alleles (ne), the Shannon index (I), and the genetic similarity and Nei distances [19]. Allelic diversity and discriminatory power of the markers were described by polymorphism information content (PIC), resolving power (Rp), and marker index (MI) and calculated using available PC software or online resource iMEC [20]. The genetic similarity dendrogram was compiled by the UPGMA method using the NTSYSpc v2.10 software [21]; GenAlEx 6.5 software was used to determine molecular dispersion and PCoA analysis [22, 23].

## Results

**Fig. 1:**
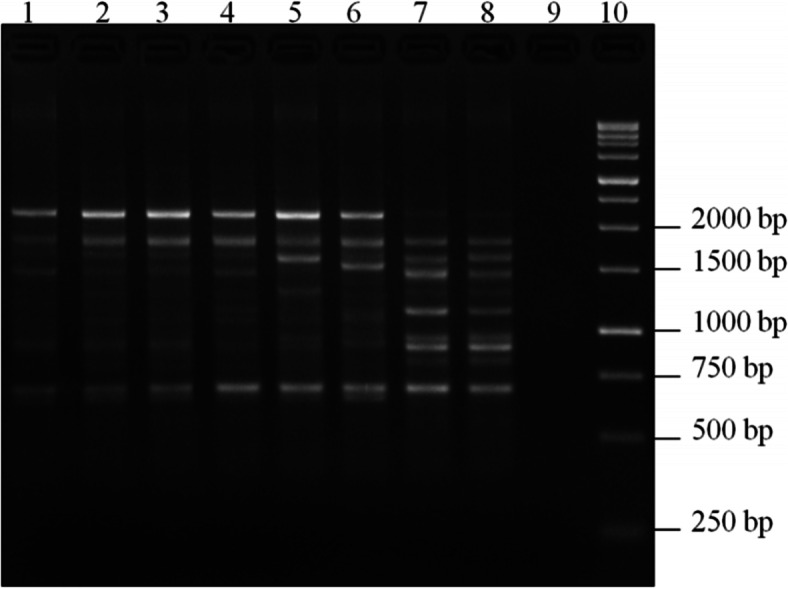
Electropherogram of PCR products of ryegrass samples with SCoT-06 marker. Perennial ryegrass cultivars: Agat (1), Duet (2), VIK 66 (3), Karat (4), Fenix (5), VIK 22 (6); annual ryegrass: Rapid (7), Moskovsky 74 (8); 9 – control (H_2_O); 10 – molecular weight marker (1kb DNA Ladder, Evrogen, Russia)

The set of 25 SCoT primers was used for genotyping of ryegrass samples, consisting of 30 genotypes per cultivar. More informative of them, generating distinct and reproducible amplification fragments, were selected for DNA polymorphism estimation (Fig. 1).

A total of 107 PCR products were obtained with an average of 13 amplicons per one marker. The amplified DNA fragments ranged from 349 to 2718 b.p. (Table 3). In analyzed ryegrass samples, we observed a moderate polymorphism level of 34.6%. The effective numbers of alleles (ne) were in different ranges for different Scot markers, wherein the least value range was for the marker SCoT 32 (1.35) and the highest was for SCoT 02 (1.58), with an average 1.48 per locus. A marker SCoT-06 had the highest polymorphism level (50%) in comparison to other ones. We determined the Polymorphism Information Content (PIC) value that is an indicator of the markers’ power. The highest PIC value (0.37) was present for SCoT 02, SCoT 23, SCoT 32, and SCoT 35, and the lowest (0.29) was characteristic for the marker SCoT 06. The average Shannon index (I) was 0.46 (see Table 3). According to the results obtained, marker index (MI) was in different ranges depending on the used SCoT markers, wherein the least value range was for SCoT 06 (0.01) and the highest was for SCoT 32 (0.04). The marker SCoT 13 had the maximum rate of Rp (resolving power), which amounted to 10.

**Table 3:** Indicators of genetic variability and information content of SCoT markers based on the analysis of Russian ryegrass cultivars

No.	Marker	Effective number of alleles (ne)	Shannon index (I)	Polymorphism level (%)	The indicator of the primer informativeness (PIC)	Resolving power (Rp)	Marker index (MI)
1	SCoT 02	1.58	0.53	20.0	0.37	2.5	0.03
2	SCoT 20	1.53	0.49	26.7	0.36	7	0.02
3	SCoT 23	1.37	0.38	25.0	0.37	1.25	0.03
4	SCoT 06	1.45	0.47	50.0	0.29	9.25	0.01
5	SCoT 13	1.52	0.50	40.9	0.33	10	0.02
6	SCoT 32	1.35	0.32	20.0	0.37	3	0.04
7	SCoT 22	1.50	0.48	45.5	0.35	5	0.02
8	SCoT 35	1.56	0.51	18.8	0.37	7.75	0.03
	Average	1.48	0.46	30.9	0.35	5.72	0.03

The indices of genetic similarity and Nei’s genetic distances were evaluated based on polymorphism data using simple match coefficient (SM) and NTSYSpc v2.02 software (Table 4). The minimum value of genetic distance (0.39) was found among ryegrass cultivars Agat and Rapid. The highest value (0.88) was determined for Rapid and Moscovsky 74.

**Table 4:** Indices of genetic similarity (above the diagonal) and Nei distances (under the diagonal)

Cultivar^a^	AG	DT	V6	KT	FX	V2	RD	M74
AG	****	0.79	0.73	0.73	0.66	0.63	0.39	0.40
DT	0.24	****	0.86	0.81	0.77	0.70	0.41	0.45
V6	0.32	0.15	****	0.85	0.76	0.69	0.45	0.46
KT	0.32	0.21	0.16	****	0.83	0.70	0.45	0.48
FX	0.42	0.26	0.27	0.19	****	0.75	0.49	0.52
V2	0.46	0.36	0.37	0.35	0.29	****	0.50	0.51
RD	0.93	0.89	0.80	0.80	0.71	0.69	****	0.88
M74	0.91	0.80	0.78	0.74	0.66	0.68	0.13	****

^a^The names of cultivars are represented by an abbreviation (see Table 1)

**Fig. 2:**
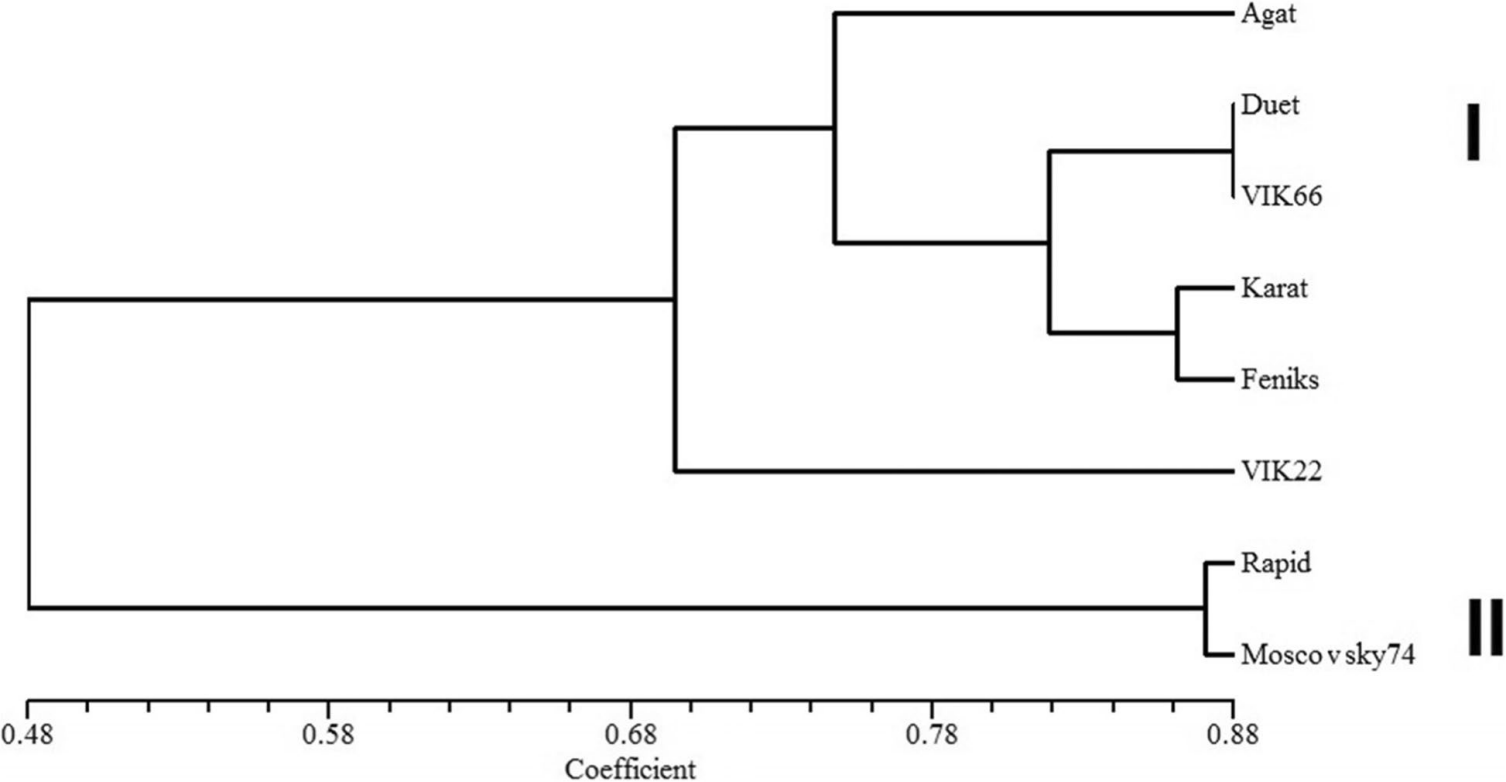
UPGMA dendrogram of ryegrass cultivars based on the results of genotyping using SCoT markers: I – the first cluster; II – the second cluster

We used the Unweighted Pair Group Method with Arithmetic Mean (UPGMA) to investigate the relationships between the different samples. The cluster analysis and UPGMA dendrogram arranged the samples in the two groups (Fig. 2). The first cluster contains following cultivars: Agat, Duet, VIK 66, Karat, Feniks, VIK 22. The second cluster comprises accessions Rapid and Moscovsky 74. Grouping of samples was in accordance with species differences and their ploidy level.

**Fig. 3:**
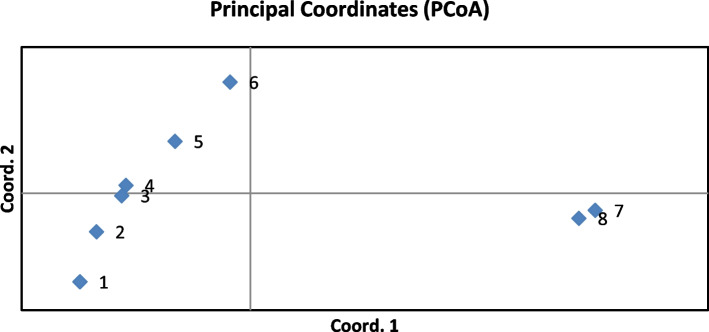
PCoA analysis of the results of genotyping ryegrass cultivars using SCoT markers: 1 –Agat; 2 – Duet; 3 – VIK 66; 4 – Karat; 5 – Fenix; 6 – VIK 22; 7 – Rapid; 8 –Moskovsky 74

Figure 3 refers to the PCoA (principal coordinate analysis) of tested cultivars, based on SCoT markers and it shows that the values of the first and second coordinates were 48.7% and 14.9% respectively and explained 63.6% of the total genetic variability of the original data. The results of PCoA analysis were consistent with the results obtained on the basis of cluster analysis.

In addition, analysis of molecular variance (AMOVA) was conducted to study cultivars’ genetic variations based on 25 SCoT markers. AMOVA showed significant interspecies differentiation (59%), while between cultivars within groups, this indicator was 41%.

## Discussion

Cross-pollinated species, which include ryegrass, are characterized by a high degree of genetic variability between and within populations; therefore, an increase in the number of individuals included in the general sample increases the reliability of the obtained results. For ryegrass, the sample from a cultivar or population should include at least 24 genotypes to provide the coverage of the most common alleles [24]. In our work, the analyzed samples, representing ryegrass cultivars of 2 species, consisted of 30 genotypes from each cultivar. The use of “bulk samples” significantly reduced the time, labor, and financial cost of maintaining the acceptable size of the accessions for analysis.

The total number of obtained PCR products and the level of polymorphism were compatible with the results, obtained in the research studies of other authors. So, Tabaripour and Keshavarzi [8] at genotyping of 50 ryegrass samples of different species with SCoT markers showed that the level of polymorphism in *Lolium perenne* L. equaled 46.88% while in *Lolium multiflorum* Lam. − 34.88%.

The average number of alleles per locus (13) and effective number of alleles (averaged 1.48) in our study was a significant indicator of the genetic diversity of investigated samples and it was higher than the values obtained by Cheng et al. [25] when analyzing 19 samples of the genus *Lolium* L. using SRAP markers (effective number of alleles was – 1.27). Other indicators of genetic diversity, determined according to the results of our studies, such as PIC values (0.35) and Shannon’s index (0.46 versus 0.24) were also somewhat higher.

Analysis of molecular variance (AMOVA) was used to test genetic variation among the studied ryegrass cultivars. We expected the high level of differentiations because of the cross-pollination of this specie and the nature of the cultivars: most of them were developed as complex-hybrid variety populations. The obtained data on the ratio of genetic variability degree corresponds to the results, represented by Tabaripour and Keshavarzi [8], although the quantitative indicators differ significantly: 71% and 29%, respectively. At the same time, Farshadfar et al. [26] found a lower level of genetic polymorphism, generated by SCoT markers, between species than among samples within species (49%).

The clustering results divided ryegrass cultivars into two groups. In the first cluster tetraploid accessions, mostly obtained by polyploidization based on wild or old local diploid varieties, were located. For example, VIK 66 and Duet were derived using the original wild-growing forms from Karelia, Tambov, and Leningrad regions. The closeness of these cultivars on the dendrogram was probably due to the presence of common sources of initial material during the breeding process. Annual ryegrass Rapid was also obtained by polyploidization with colchicine treatment based on the Moskovsky 74 cultivar. These two cultivars were located in the second cluster of the dendrogram at a close genetic distance. Despite the similarity in genetic status, they differ significantly in morphological characteristics and productivity, which is 25-30% higher in the Rapid cultivar.

When developing new cultivars of ryegrass, Russian breeders often use the method of creating synthetic hybrid populations based on crossing forms that are similar in morphological characteristics, but originating from different geographic zones. The number of initial forms during free hybridization can exceed tens. A characteristic feature of populations created in this way is the maintaining of heterosis effect for 4–5 generations and a high degree of intrapopulation variability. The cultivars Karat and Fenix were obtained by the method of complex-hybrid populations and located on the common branch of the UPGMA dendrogram.

The performed PCoA analysis clearly demonstrates the division of the studied cultivars by species differences, as was observed during the cluster analysis, except that the PCoA allocated separately VIC 22 accession in the group of perennial ryegrass. This cultivar was obtained from the hybridization of breeding samples from the central part of Russia (Tambov region) and wild-growing forms from the Baltic. It is characterized by high adaptive plasticity, resistance to frequent and low mowing, and is suitable for lawn use. The morphological and biological characteristics of cereal grasses for different purposes are often accompanied by varying degrees of genetic variability, which are evident in the works of other researchers. For example, Bolaric et al. [6] reported that the data of analyzing perennial ryegrass (22 European cultivars) using RAPD markers, allowed to cluster them depending on the fodder or lawn purpose.

Pairwise comparative analysis of genetic relationship indexes revealed the greatest genetic similarity between perennial ryegrass cultivars VIK 66 and Duet (0.86). On the contrary, the most distant were VIK 22 and Agat, with a value of the genetic distance of 0.46. According to the results of the clustering and indicators of Nei’s genetic distances (0.32), a close relationship between Agat and VIK 66 cultivars was not found, despite the common origin. Agat cultivar was obtained from VIK 66 on the experimental plot with an artificial infection loading by selecting the forms with high disease resistance. One of the reasons for the genetic remoteness may be explained by the high level of intravarietal polymorphism revealed during the genotyping of individual plants within Agat cultivar (37%). Besides, it is possible that some of the common rare alleles, presented in both cultivars, were dropped out of analysis and calculation due to the use of a total DNA probe for each sample, consisting of different genotypes (“drop-up alleles”) [27, 28].

## Conclusion

Molecular and genetic analysis of the major species of ryegrass using SCoT markers was carried out for the first time in Russia. In studying the genetic variability of varietal material, we tested the potential of the new technology.

The research results indicate the high efficiency of SCoT markers for the analysis of complex highly heterogeneous ryegrass cultivar populations. Using markers of this type, it was possible to assess the level of polymorphism between cultivars of perennial and annual ryegrass, which amounted to 38.1% and 42.8%, respectively, with an average value of 30.8%. The indicators of the primer informativeness included in the analysis varied from 0.29 (SCoT 32) to 0.37 (SCoT 02, SCoT 23, SCoT 32, SCoT 35. Markers have been identified that can serve to identify cultivars within the tested sample. Thus, using the SCoT 13 marker, it was possible to obtain unique DNA profiles for 3 cultivars at once: VIK 66, Karat, and Duet. The SCoT 02 marker turned out to be suitable for differentiating the cultivars of the one-year-old ryegrass Rapid and Moskovsky 74, and the SCoT 06, SCoT 22, and SCoT 35 markers — for the Fenix, Agat and VIK 22 cultivars, respectively.

The level of intravarietal polymorphism revealed in our study turned out to be higher than the differences between cultivars, which indicates a significant heterogeneity of the varietal material and corresponds to the data on the sources and methods of its production. At the same time, a high degree of genetic diversity, due to the heterogeneous nature of the culture, can serve as an important factor in the success of breeding programs.

Thus, it was found that SCoT markers are highly polymorphic and have the necessary discriminatory potential for distinguishing between species and cultivars of Russian ryegrass breeding. The research results are of practical importance for use in varietal identification and inbreeding cultivars for different purposes adapted to the various environmental conditions of the Russian Federation.

## Data Availability

All data generated or analyzed during this study are included in this published article.

## Abbreviations

PCoA: Principal coordinate analysis
SCoT: Start codon targeted
UPGMA: Unweighted pair group method with arithmetic mean algorithm
*H*: Nei’s gene diversity
*I*: Shannon index
Ne: Number of effective alleles
PIC: Polymorphism information content
AMOVA: Analysis of molecular variance
